# Microparticles as antigenic targets in human and murine SLE

**DOI:** 10.1186/ar3589

**Published:** 2012-02-09

**Authors:** Anirudh Ullal, David S Pisetsky

**Affiliations:** 1 Duke University Medical Center, Durham, NC, USA; 2 Durham VAMC, Durham, NC, USA

## 

Microparticles are small membrane-bound vesicles that are released from activated and dying cells by a blebbing process. These particles circulate in the blood and display potent pro-inflammatory and pro-thrombotic activities. In addition, particles are an important source of extracellular DNA and RNA and may participate in the transfer of informational nucleic acids. Because microparticles contain DNA as well as other nuclear antigens, we have investigated their ability to bind to anti-DNA and other anti-nuclesome antibodies that characterize the prototypic autoimmune disease systemic lupus erythematosus (SLE). For this purpose, we generated microparticles from HL-60, Jurkat and THP-1 cells induced to undergo apoptosis *in vitro*. Using FACS analysis to assess antibody binding, we showed that particles can bind some but not all monoclonal anti-DNA and anti-nucleosome antibodies from MRL-*lpr/lpr *and NZB/NZWF1 lupus mice. For the monoclonal anti-DNA, DNase treatment reduced binding. Like the monoclonal antibodies, patient plasma also bound to the particles although this activity was not directly correlated with levels of anti-DNA antibodies as measured by an ELISA. To determine whether particles circulating in the blood of patients can represent immune complexes, FACS analysis was performed on particles isolated from patient plasma. These studies indicated that, while the total levels of microparticles in the blood of patients with SLE did not differ significantly from those of normal controls, the number of IgG-positive particles was significantly elevated using a R-phycoerythrin-labeled anti-human IgG (γ-chain specific) reagent. In this study, the number of IgG-positive particles was correlated with levels of anti-DNA. In similar studies with plasma from MRL-*lpr/lpr *and NZB/NZWF1 mice, we showed that the total levels of particles were increased compared to those of BALB/c control mice and that the number of particles that stained with an anti-IgG reagent was also increased. Furthermore, plasma of mice could bind to particles generated *in vitro *from apoptotic cells. Together, these findings indicate that microparticles can express antigenically active DNA in an accessible form, either because of a surface location or particle permeability. Furthermore, they demonstrate that microparticles can form immune complexes and that at least some of the immune complexes in the blood in SLE contain particles. Current studies are characterizing the immune properties of these complexes and their potential role in pathogenicity.

